# Correction: Cruciani et al. Ivermectin for Prophylaxis and Treatment of COVID-19: A Systematic Review and Meta-Analysis. *Diagnostics* 2021, *11*, 1645

**DOI:** 10.3390/diagnostics11122359

**Published:** 2021-12-14

**Authors:** Mario Cruciani, Ilaria Pati, Francesca Masiello, Marina Malena, Simonetta Pupella, Vincenzo De Angelis

**Affiliations:** 1 Italian National Blood Centre, National Institute of Health, 00162 Rome, Italy; ilaria.pati@iss.it (I.P.); francesca.masiello@iss.it (F.M.); simonetta.pupella@iss.it (S.P.); vincenzo.deangelis@iss.it (V.D.A.); 2 Infectious Diseases Unit, AULSS9 Scaligera, 37100 Verona, Italy; marina.malena@aulss9.veneto.it

## Affiliation Correction

The correct affiliation 1 should be “Italian National Blood Centre, National Institute of Health, 00162 Rome, Italy”.

## Missing Citation

In the original article, “Schünemann, H.J.; Oxman, A.D.; Higgins, J.P.; Vist, G.E.; Glasziou, P.; Guyatt, G.H. Chapter 11: Presenting results and ‘Summary of findings’ tables. In *Cochrane Handbook for Systematic Reviews of Interventions Version 5.1.0 (Updated March 2011)*; Higgins, J.P., Green, S., Eds.; The Cochrane Collaboration, 2011. Available online: handbook.cochrane.org (accessed on 1 June 2021)“ was not cited. The citation has now been inserted in Section 2.6. Assessment of Risk of Bias in Included Studies, and should read:

Two review authors (I.P., M.C.) independently assessed the risk of bias of each study included following the domain-based evaluation described in the Cochrane Handbook for Systematic Reviews of Interventions (Available from www.handbook.cochrane.org, accessed on 18 August 2021) [33,34]. 

## Text Correction

When submitting in August 2021 our manuscript [1] to *Diagnostics*, we were not aware that one of the trials included in the review, an Egyptian study of ivermectin for COVID-19 by Elgazzar et al. published as preprint in “Research Square” [2], had been retracted over concerns of plagiarism and serious problems with their raw data, as reported in a press release on “The Guardian” and then on “BBC news” [3,4]. Research Square withdrew this preprint on 14 July, when they were presented with “evidence of both plagiarism and anomalies in the dataset associated with the study, neither of which could reasonably be addressed by the author issuing a revised version of the paper”.

Subsequently, Open Forum Infectious Diseases, an official journal of the Infectious Diseases Society of America published an expression of concern. This has prompted other authors of systematic reviews and meta-analysis of ivermectin for COVID-19 to retract the published paper [3], and to plan the submission of a revised version of the paper, excluding the “Fraudulent” study [5].

In our systematic review [1], we performed a methodological assessment of the included trials using an appropriate check list for risk of bias (ROB for RCTs, and ROBIN-1 for non RCTs) and GRADE assessment, as required by the new Cochrane standards. The certainty of the available evidence was graded as low or very low, considering the risk of bias in the majority of the included studies (including that of Elgazzar et al.), the imprecision (reflecting the inadequate numbers of participants and/or events) and the inconsistency (heterogeneity). We concluded that “there is limited evidence for the benefit of ivermectin for COVID-19 treatment and prophylaxis, and most of this evidence is of low quality”. Certainly, one of the limitations of our review and other reviews on COVID-19 treatments is related to the fact that many of the data continue to be published in preprints and protocol repositories, which do not follow the recommended processes to ensure high quality standards for publications.

We did not perform sensitivity analyses for the outcomes analysed, also considering that the take home message was about the limited evidence of benefit of ivermectin compared to standard treatment. Considering all of this, we have now performed a sensitivity analysis excluding the study by Elgazzar et al. The take home message of our paper is almost the same, and the only relevant difference after exclusion of this paper is that the difference in the occurrence of mortality in the subgroup of patients with severe baseline conditions is no longer favouring ivermectin compared to controls, as shown below (Table 1 and Figure 1, forest plots of comparisons). As in the previous analyses, the certainty of the available evidence remains low, which means that further research is very likely to have an important impact on the confidence in the estimation of effects and is likely to change the estimate, regardless of the fact that the study by Elgazzar et al. is included or not in the analyses. Therefore, there is no need for a new systematic review and meta-analysis, but just a fine-tuning before and after the exclusion of this study.

In conclusion, the available evidence continues to be not adequate to support the use of ivermectin for the treatment of COVID-19 in clinical practice. However, more studies are underway, and it would be better wait further evidence before concluding that ivermectin has no place in COVID-19 treatment.

The following paragraph is added in the original publication (Section 3.8. Mortality, page 11, after the paragraph “When the analysis was restricted to studies or subsets of patients with baseline severe diseases …”):

In sensitivity analysis, after the exclusion of the study by Elgazzar et al. [35] in the subset of studies with baseline severe conditions the difference in the occurrence of mortality is not longer favouring ivermectin compared to controls (MD, −0.14 (95% CIs, −0.30/0.02; *p* = 0.08). As in the previous analyses, the certainty of the available evidence remains low.

The authors apologize for any inconvenience caused and state that the scientific conclusions are substantially unaffected.

## Figures and Tables

**Figure 1:**
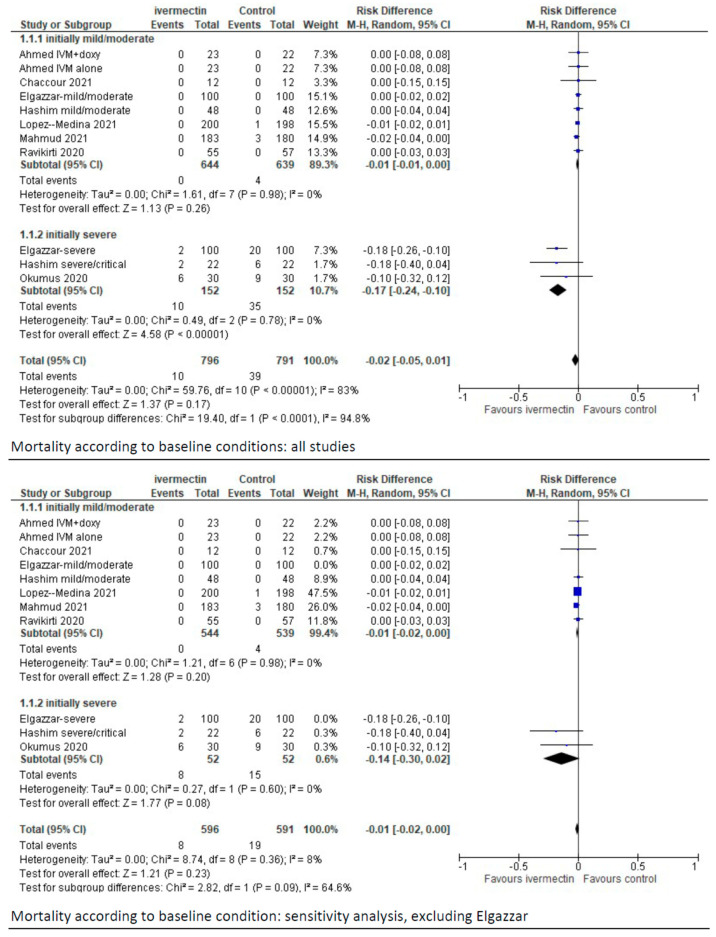
Forest plot of the comparison. Outcome: mortality according to baseline conditions. Top: all studies; bottom: sensitivity analysis excluding Elgazzar et al. [2].

**Table 1:**
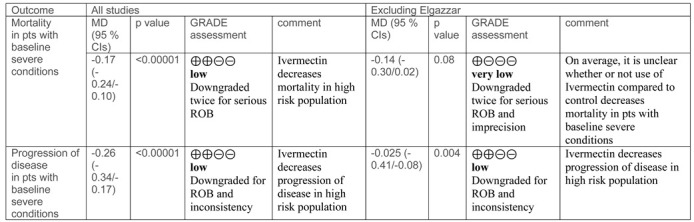
Sensitivity analysis of some outcomes of the meta-analysis.

MD, mean difference. CIs. Confidence intervals. ROB, Risk of bias.
