# Supplementary material for: Ivermectin for Prophylaxis and Treatment of COVID-19: A Systematic Review and Meta-Analysis
Source: Diagnostics (Basel). 2021 Sep 8;11(9):1645. doi: 10.3390/diagnostics11091645 (PMC8470309; doi:10.3390/diagnostics11091645)
Supplement: Supplementary file 1 [file diagnostics-11-01645-s001.zip › Suppl. Figure S1. Risk of bias graphs.pdf]

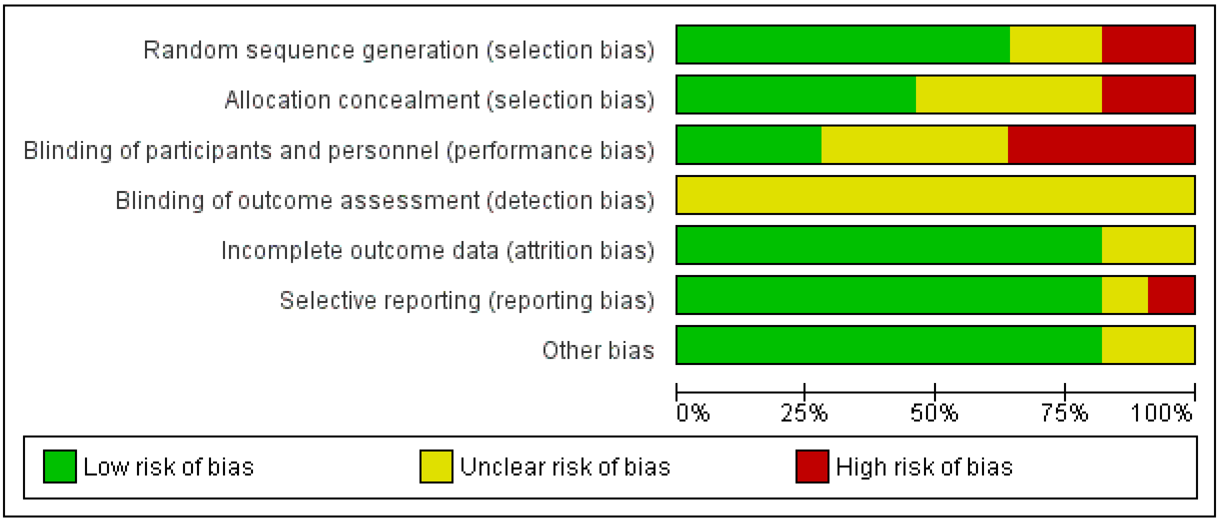

Risk of bias graph: review authors' judgements about each risk of bias item presented as percentages across all included studies.

	Random sequence generation (selection bias)	Allocation concealment (selection bias)	Blinding of participants and personnel (performance bias)	Blinding of outcome assessment (detection bias)	Incomplete outcome data (attrition bias)	Selective reporting (reporting bias)	Other bias
Ahmed 2021	?	?	?	?	+	+	+
Chaccour 2021	+	+	+	?	+	+	+
Elgazzar	+	?	?	?	?	+	+
Hashim 2020	−	−	−	?	+	+	+
IVERCAR-TUC	+	?	−	?	+	+	?
Lopez--Medina 2021	+	+	+	?	+	+	?
Mahmud 2021	+	+	+	?	+	+	+
Niaee 2020	+	+	?	?	+	?	+
Okumus 2020	−	−	−	?	+	+	+
Ravikirti 2020	+	+	?	?	+	+	+
Shoumann 2021	?	?	−	?	?	−	+

Risk of bias summary: review authors' judgements about each risk of bias item for each included study.
