# Supplementary material for: Ivermectin for Prophylaxis and Treatment of COVID-19: A Systematic Review and Meta-Analysis
Source: Diagnostics (Basel). 2021 Sep 8;11(9):1645. doi: 10.3390/diagnostics11091645 (PMC8470309; doi:10.3390/diagnostics11091645)
Supplement: Supplementary file 1 [file diagnostics-11-01645-s001.zip › Table S1. characteristic of studies and risk of bias.pdf]

Characteristics of studies

Characteristics of included studies

Ahmed 2021

**Methods**	A randomized, double-blind, placebo-controlled trial
**Participants**	The trial included 72 hospitalized patients in Dhaka, Bangladesh, The duration of illness before assessment was an average of 3.83 days
**Interventions**	three groups: oral ivermectin alone (12 mg once daily for 5 days), oral ivermectin in combination with doxycycline (12 mg ivermectin single dose and 200 mg doxycycline on day 1, followed by 100 mg every 12 h for the next 4 days), and a placebo control group
**Outcomes**	The primary endpoints were the time required for virological clearance (a negative rRT-PCR result on nasopharyngeal swab), and remission of fever (37.5 C) and cough within 7 days. Secondary outcomes included failure to maintain an SpO2 >93% despite oxygenation and days on oxygen support, the duration of hospitalization, and all-cause mortality. adverse events were also recorded
**Notes**	

Risk of bias table

**Bias**	**Authors' judgement**	**Support for judgement**
Random sequence generation (selection bias)	Unclear risk	no information provided. Study defined as RCT in methods, but not in title and abstract
Allocation concealment (selection bias)	Unclear risk	no information provided
Blinding of participants and personnel (performance bias)	Unclear risk	no information provided
Blinding of outcome assessment (detection bias)	Unclear risk	no information provided
Incomplete outcome data (attrition bias)	Low risk	One patient from each of the ivermectin + doxycycline and placebo groups and two patients in the 5-day ivermectin group withdrew their consent during the study due to family obligations and unwillingness to be tested further.
Selective reporting (reporting bias)	Low risk	all outcomes reported
Other bias	Low risk	no other potential source of bias identified

Chaccour 2021

**Methods**	A pilot, double-blind, placebo-controlled, randomized clinical trial
**Participants**	patients with non-severe COVID-19 and no risk factors for complicated disease attending the emergency room of the Clínica Universidad de Navarra between July 31, 2020 and September 11, 2020. All enrollments occurred within 72 h of onset of fever or cough.
**Interventions**	Patients were randomized 1:1 to receive ivermectin, 400 mcg/kg, single dose (n = 12) or placebo (n = 12).
**Outcomes**	The primary outcome measure was the proportion of patients with detectable SARS-CoV-2 RNA by PCR from nasopharyngeal swab at day 7 post-treatment.
**Notes**	

Risk of bias table

**Bias**	**Authors' judgement**	**Support for judgement**
Random sequence generation (selection bias)	Low risk	Eligible patients will be allocated in a 1:1 ratio using a randomization list generated by the trial statistician using blocks of four to ensure balance between the groups.
Allocation concealment (selection bias)	Low risk	A study identification code will be generated using a sequence of random numbers so that the randomization number does not match the subject identifier. The sequence and code used will be kept in an encrypted file accessible only to the trial statistician.
Blinding of participants and personnel (performance bias)	Low risk	The clinical trial team and the patients will be blinded. The placebo will not be visibly identical, but it will be administered by staff not involved in the clinical care or participant follow up
Blinding of outcome assessment (detection bias)	Unclear risk	no information provided
Incomplete outcome data (attrition bias)	Low risk	All patients recruited completed the tria
Selective reporting (reporting bias)	Low risk	all outcomes reported
Other bias	Low risk	no other potential source of bias identified

Elgazzar

**Methods**	A multicenter double blind randomized controlled clinical trial.
**Participants**	200 pts with mild/moderate COVID-19 infections, and 200 pts with severe COVID-19 infections
**Interventions**	Group I: 100 patients with mild/moderate COVID-19 infection received a 4-days course of Ivermectin plus standard of care; Group II: 100 patients with mild/moderate COVID-19 infection received hydroxychloroquine plus standard care; Group III: 100 patients with severe COVID-19

	infection received Ivermectin plus standar care; Group IV: 100 patients with Severe COVID-19 infection received hydroxychloroquine plus standard care.
**Outcomes**	The primary endpoint: clinical, laboratory investigations improvement and/or 2 consecutive negative PCR tests taken at least 48 hours apart. Secondary endpoint: Patients presenting with adverse events requiring stoppage of treatment and management of any side effects accordingly.
**Notes**	Other 200 health care and household contact were enroled in a prophylaxis study

Risk of bias table

**Bias**	**Authors' judgement**	**Support for judgement**
Random sequence generation (selection bias)	Low risk	Randomization A Block randomization method was used to randomize the study participants into two groups that result in equal sample sizes. This method was used to ensure a balance in sample size across groups over time and keep the numbers of participants in each group similar at all times.
Allocation concealment (selection bias)	Unclear risk	no information provided
Blinding of participants and personnel (performance bias)	Unclear risk	in the clin.gov protocol, the study is defined as Triple blind (Participant, Care Provider, Investigator), but no further information is provided
Blinding of outcome assessment (detection bias)	Unclear risk	as above
Incomplete outcome data (attrition bias)	Unclear risk	No information provided on the number of pts in each group that completed the study
Selective reporting (reporting bias)	Low risk	all outcomes reported
Other bias	Low risk	no other potential source of bias identified

Hashim 2020

**Methods**	Randomized controlled study
**Participants**	70 COVID-19 patients (48 mild-moderate, 11 severe, and 11 critical patients) treated with ivermectine and 70 pts (48 mild-moderate and 22 severe and zero critical patients) on standard therapy.
**Interventions**	Ivermectin 200ug/kg PO per day for 2-3 days along with 100mg PO doxycycline twice per day for 5-10 days plus standard therapy vs standard therapy (which included azithromycin and dexamethazone when required)
**Outcomes**	The time to recovery, the progression of the disease, and the mortality rate were the outcome-assessing parameters.

**Notes**	adverse events not considered

Risk of bias table

Bias	Authors' judgement	Support for judgement
Random sequence generation (selection bias)	High risk	Patients recruited at dates with odd number were allocated Ivermectin-Doxycycline group while other patients were allocated to the control group
Allocation concealment (selection bias)	High risk	Patients recruited at dates with odd number were allocated Ivermectin-Doxycycline group while other patients were allocated to the control group
Blinding of participants and personnel (performance bias)	High risk	open label
Blinding of outcome assessment (detection bias)	Unclear risk	the randomization process as well as the patients records for disease progression, recovery, and clinical or laboratory testing were supervised by the health authority of Alkarkh Health General Directorate in Baghdad city.
Incomplete outcome data (attrition bias)	Low risk	all pts completed the study
Selective reporting (reporting bias)	Low risk	all outcomes reported
Other bias	Low risk	no other potential source of bias identified

IVERCAR-TUC

**Methods**	A RCT, open label
**Participants**	234 health care personnel (medical personnel, nurses,kinesiologists) and also administrative and cleaning personnel
**Interventions**	The experimental group received Ivermectin orally 2 tablets of 6 mg = 12 mg every 7 days, and the control group Iota-Carrageenan 6 sprays per day for 4 weeks
**Outcomes**	A post-control follow-up was carried out at 14 days (remote clinical telemedicine follow-up) at the end of which an RT-PCR test was performed. Subjects were evaluated every 7 days in 4 visits from the beginning of the study. Enrolled subjects completed symptom questionnaires (including reporting any adverse effects of treatmen), physical examinations, and COVID-19 nasopharyngeal secretion tests (RT PCR) at each time
**Notes**	

Risk of bias table

Bias	Authors' judgement	Support for judgement

Random sequence generation (selection bias)	Low risk	The selection to each group was performed through a random number generation process by an Excel spreadsheet.
Allocation concealment (selection bias)	Unclear risk	no information provided
Blinding of participants and personnel (performance bias)	High risk	open label
Blinding of outcome assessment (detection bias)	Unclear risk	no information provided
Incomplete outcome data (attrition bias)	Low risk	all pts completed the study
Selective reporting (reporting bias)	Low risk	all outcomes reported
Other bias	Unclear risk	a lower proportion of non-health care personnel was enrolled in the IVM group compared to house hold contact (15.3 vs 29.9 %)

Lopez--Medina 2021

**Methods**	Double-blind, randomized trial
**Participants**	A total of 476 adult patients with mild disease and symptoms for 7 days or fewer (at home or hospitalized) were enrolled between July 15 and November 30, 2020, and followed up through December 21, 202.
**Interventions**	Patients were randomized to receive ivermectin, 300 μg/kg of body weight per day for 5 days (n = 200) or placebo (n = 200)
**Outcomes**	Primary outcome was time to resolution of symptoms within a 21-day follow-up period. Solicited adverse events and serious adverse events were also collected
**Notes**	conducted at a single site in Cali, Colombia. Potential study participants were identified by simple random sampling from the state's health department electronic database of patients with symptomatic, laboratory-confirmed COVID-19 during the study period.

Risk of bias table

**Bias**	**Authors' judgement**	**Support for judgement**
Random sequence generation (selection bias)	Low risk	simple random sampling from the state's database. Patients were randomized in permuted blocks of 4 in a randomization sequence prepared by the unblinded pharmacist in Microsoft Excel version 19.0
Allocation concealment (selection bias)	Low risk	the pharmacist provided masked ivermectin or placebo to a field nurse for home or hospital patient visits.
Blinding of participants and	Low risk	Allocation assignment was concealed from investigators and patients.Because blinding could be jeopardized due to the

personnel (performance bias)		different taste and smell of ivermectin and the saline/dextrose placebo, only 1 patient per household was included in the study until the manufacturer's placebo was available. Bottles of ivermectin and placebo were identical throughout the study period to guarantee double-blinding.
Blinding of outcome assessment (detection bias)	Unclear risk	A study physician reviewed medical records of hospitalized patients to obtain the information requireld by the protoco
Incomplete outcome data (attrition bias)	Low risk	No data were missing for the primary or secondary outcomes.
Selective reporting (reporting bias)	Low risk	all outcomes reported
Other bias	Unclear risk	the study was not conducted or completed according to the original design, and the original primary outcome to detect the ability of ivermectin to prevent clinical deterioration was changed 6 weeks into the trial. In the study population, the incidence of clinical deterioration was below 3%, making the original planned analysis futile

Mahmud 2021

**Methods**	randomized, blinded, placebo-controlled trial
**Participants**	patients with mild-tomoderate COVID-19 symptoms r
**Interventions**	The treatment group received a single dose of ivermectin 12 mg and doxycycline 100 mg, twice daily for 5 days, in addition to standard of care. Standard of care included administration of paracetamol, antihistamines, cough suppressants, vitamins, oxygen therapy according to indication and need, low molecular weight heparin according to indication, appropriate other broad-spectrum antibiotics, remdesivir injection, other antiviral drugs, and other drugs for associated comorbid conditions. The placebo group received placebo in addition to standard of care.
**Outcomes**	The primary outcome was duration from treatment to clinical recovery. Secondary outcomes were disease progression and persistent COVID-19 positivity by RT-PCR. Adverse events were also recorded.
**Notes**	

Risk of bias table

**Bias**	**Authors' judgement**	**Support for judgement**
Random sequence generation (selection bias)	Low risk	The allocation schedule was created with a list of random numbers generated using a random number generator program by the head of the Department of Medicine of Dhaka Medical College.

Allocation concealment (selection bias)	Low risk	Group assignment was concealed in sequentially numbered, opaque, sealed envelopes. The randomization code was maintained by the pharmaceutical company.
Blinding of participants and personnel (performance bias)	Low risk	Both the investigators and the patients were blinded to the treatment allocation
Blinding of outcome assessment (detection bias)	Unclear risk	The coinvestigators assessed the outcome, graded the disease, and documented adverse reactions
Incomplete outcome data (attrition bias)	Low risk	Among the 200 patients in the placebo group, 17 were lost to follow-up, 3 died, and 180 completed the follow-up. Among the 200 patients in the treatment group, 15 were lost to follow-up, 2 discontinued owing to adverse effects, and 183 completed follow-up. Intention-to-treat analysis was performed. . Details of patients who were lost to follow-up, had died, or had withdrawn from the trial owing to adverse effects were censored on the final study day.
Selective reporting (reporting bias)	Low risk	all outcomes reported
Other bias	Low risk	no other potential source of bias identified

Niaee 2020

**Methods**	A 45-days randomized, double-blind, placebo-controlled, multicenter, phase 2 clinical trial
**Participants**	A total number of 180 mild to severe hospitalized patients with COVID-19.
**Interventions**	All patients were treated according to “Iranian guideline of hospitalized COVID-19 patients’ management (version 5)”. This comprised oral hydroxychloroquine (HCQ) 200mg/kg twice per day as standard regimen and a heparin prophylaxis in combination with supplemental oxygen. The participants were randomly allocated to six arms including standard regimen (Hydroxychloroquine 200mg/kg twice per day), placebo plus standard regime, single dose ivermectin (200mcg/Kg, 1 pill per day), three low interval doses of ivermectin (200, 200, 200 mcg/Kg , 3 pills in 1, 3 and 5 interval days), single dose ivermectin (400mcg/Kg, 2 pills per day), and three high interval doses of ivermectin (400, 200, 200 mcg/Kg, 4 pills in 1, 3 and 5 interval days).
**Outcomes**	The primary endpoint of this trial was clinical recovery within 45 days of enrolment
**Notes**	

Risk of bias table

**Bias**	**Authors' judgement**	**Support for judgement**

Random sequence generation (selection bias)	Low risk	Randomization was performed using Random Allocation Software,.according to the severity of the disease was as follows: mild, moderate, and severe
Allocation concealment (selection bias)	Low risk	The transposed block randomization sequence, including stratification was prepared by a statistician not involved in the trial The patients in six treatment arms enrolment were randomized after calling the central randomization telephone number and receiving randomization information and confirmation. Each patient received the unique patient numbers that were to be used on all study medication containers, case report forms, and to identify all specimens. Pharmacia generated the randomization list and provided the list to the central randomization service.
Blinding of participants and personnel (performance bias)	Unclear risk	defined as double blind, but no further information provided
Blinding of outcome assessment (detection bias)	Unclear risk	no information provided
Incomplete outcome data (attrition bias)	Low risk	all pts completed the study
Selective reporting (reporting bias)	Unclear risk	the outcomes reported were duration of hospital stay (mean and range) and mortality
Other bias	Low risk	no other potential source of bias identified

Okumus 2020

**Methods**	RCT, open label
**Participants**	Patients who were hospitalised with a pre-diagnosis of severe COVID-19 pneumonia and thereafter diagnosis of COVID-19 was also confirmed microbiologically with polymerase chain reaction (PCR) positivity in respiratory tract samples were included into the study. T
**Interventions**	Hydroxychloroquine, favipiravir and azithromycin (HFA) standard treatment protocol were given to the control group. In addition to HFA treatment, ivermectin 200 micrograms/kg/day (9mg between 36-50 kg, 12mg between 51-65 kg, 15mg between 66-79 kg and 200 micrograms/kg in > 80 kg) in the form of a solution prepared for enteral use was added (HFA+I) to the treatment protocol of the study group's for five days.
**Outcomes**	Rate of COVID-19 Polymerase Chain Reaction (PCR) Test Negativity; mortality; adverse events;; clinical response; changes in clinical and laboratory parameters
**Notes**	

Risk of bias table

Bias	Authors' judgement	Support for judgement
Random sequence generation (selection bias)	High risk	They were randomized to the study and control group, respectively. Single numbered patients were accepted as study group and double numbered patients as control group
Allocation concealment (selection bias)	High risk	allocation easily predictable
Blinding of participants and personnel (performance bias)	High risk	open label
Blinding of outcome assessment (detection bias)	Unclear risk	no information provided
Incomplete outcome data (attrition bias)	Low risk	At the beginning of the study, it was planned to have 30 patients each in the control and study groups. During the study, 6 patients were excluded from the study group because ivermectin treatments were terminated due to the detection of mutations that impairs ivermectin metabolism and new patients were added. As a result, 66 patients were included in the study, 6 patients were excluded due to mutation detection and the study was completed with 30 patients in both groups.
Selective reporting (reporting bias)	Low risk	all the outcomes reported
Other bias	Low risk	no other potential source of bias detected

Ravikirti 2020

**Methods**	RCT, DB
**Participants**	covid-19 pts with mild-moderate ilness
**Interventions**	ivermectine or placebo, but in both grpups all pts received also oh-cloroquine, steroids, >90 % enoxiparine, and also remdesivir (20 %), convalescent plasma (10 %) and other drugs
**Outcomes**	primary: negative PCT test at days 6; secondary: symptoms status at days 6; discharge status ; admission to ICU; need mechanical ventilation; death
**Notes**	

Risk of bias table

Bias	Authors' judgement	Support for judgement
Random sequence generation (selection bias)	Low risk	generated using sealed envelope software

Allocation concealment (selection bias)	Low risk	allocated on envelope
Blinding of participants and personnel (performance bias)	Unclear risk	no information provided
Blinding of outcome assessment (detection bias)	Unclear risk	no information provided
Incomplete outcome data (attrition bias)	Low risk	of the 115 pts enrolled in the study, 112 were included in the final analysis
Selective reporting (reporting bias)	Low risk	all the outcomes reported
Other bias	Low risk	no other potential source of bias detected

Shoumann 2021

**Methods**	A prospective interventional randomised open label-controlled study
**Participants**	asymptomatic family close contacts with COVID-19 patients
**Interventions**	In ivermectin arm, contacts received ivermectin according to Body Weight (BW) on day of the diagnosis of their index case. The non-intervention group received no treatment. Group one (ivermectin group) contacts received ivermectin on the day of the diagnosis of their index case. Ivermectin was given at day one (diagnosis day) and repeated once more at day 3 (total 2 doses). The dose was adjusted according to Body Weight (BW) as follows: 15 mg/day for subjects of 40-60 kg BW; 18 mg/day for 60-80 kg; and 24 mg/day for those >80 kg BW. Regarding second (non-intervention) group, none of family members received ivermectin.
**Outcomes**	Both groups were followed-up for two weeks for development of symptoms suggestive of COVID-19. RT-PCr test for Covid-19. including fever with respiratory symptoms plus or minus others symptoms. Follow-up sheet was administered for both the managing physician and contacts. If any contact developed symptoms suggestive of COVID-19, Complete Blood Count (CBC) and C-Reactive Protein (CRP) were done just after onset of symptoms along with a High-Resolution Computed Tomography (HRCT) of the chest within 3-5 days was performed.
**Notes**	It was planned to include contacts of 50 RT-PCR confirmed COVID-19 patients in each arm. But during recruitment and as the trial was non-blinded, the high protective efficacy detected for ivermectin made the researchers to stop prematurely the non-intervention arm

Risk of bias table

**Bias**	**Authors' judgement**	**Support for judgement**
Random sequence generation (selection bias)	Unclear risk	no information provided

Allocation concealment (selection bias)	Unclear risk	no information provided
Blinding of participants and personnel (performance bias)	High risk	open label.
Blinding of outcome assessment (detection bias)	Unclear risk	no information provided
Incomplete outcome data (attrition bias)	Unclear risk	36 subjects (25/228 in IVM group and 11/112 in control group) out of 340 enrolled (which is around 10 %) did not complete the study
Selective reporting (reporting bias)	High risk	While clinical evaluation was performed in all subject included in the study, due to limitation of performing RT-PCR for suspected COVID-19 patients, only four subjects in ivermectin group and 12 subjects in the non-intervention group performed it and were positive for SARS-CoV-2. Hence, it is possible that asymptomatic infections among contacts in both groups have been missed
Other bias	Low risk	no other potential source of bias detected
