# Supplementary material for: Ivermectin for Prophylaxis and Treatment of COVID-19: A Systematic Review and Meta-Analysis
Source: Diagnostics (Basel). 2021 Sep 8;11(9):1645. doi: 10.3390/diagnostics11091645 (PMC8470309; doi:10.3390/diagnostics11091645)
Supplement: Supplementary file 1 [file diagnostics-11-01645-s001.zip › Table S2. Data and analyses.pdf]

Data and analyses

1 ivermectine vs control

Outcome or Subgroup	Studies	Participants	Statistical Method	Effect Estimate
1.1 mortality according to baseline conditions	11	1587	Risk Difference (M-H, Random, 95% CI)	-0.02 [-0.05, 0.01]
1.1.1 initially mild/moderate	8	1283	Risk Difference (M-H, Random, 95% CI)	-0.01 [-0.01, 0.00]
1.1.2 initially severe	3	304	Risk Difference (M-H, Random, 95% CI)	-0.17 [-0.24, -0.10]
1.2 All cause mortality	12	1767	Risk Difference (M-H, Random, 95% CI)	-0.04 [-0.08, -0.00]
1.2.1 ivermectin alone vs control	3	467	Risk Difference (M-H, Random, 95% CI)	-0.00 [-0.02, 0.01]
1.2.2 Ivermectin in combination vs control	9	1300	Risk Difference (M-H, Random, 95% CI)	-0.06 [-0.11, -0.00]
1.3 viral clearance (% patients)	7		Risk Difference (M-H, Random, 95% CI)	Subtotals only
1.3.1 at 6-10 days	6	430	Risk Difference (M-H, Random, 95% CI)	0.10 [-0.12, 0.31]
1.3.2 at 14 days	3	453	Risk Difference (M-H, Random, 95% CI)	0.21 [0.05, 0.36]
1.4 disease progression	11	1707	Risk Difference (M-H, Random, 95% CI)	-0.09 [-0.16, -0.02]
1.4.1 initially mild/moderate	8	1405	Risk Difference (M-H, Random, 95% CI)	-0.05 [-0.11, 0.00]
1.4.2 initially severe	3	302	Risk Difference (M-H, Random, 95% CI)	-0.26 [-0.34, -0.17]

Outcome or Subgroup	Studies	Participants	Statistical Method	Effect Estimate
1.1 Adverse events	7		Risk Difference (M-H, Random, 95% CI)	Subtotals only
1.1.1 Overall AE	5	1724	Risk Difference (M-H, Random, 95% CI)	-0.01 [-0.11, 0.08]
1.1.2 Serious AE	6	1428	Risk Difference (M-H, Random, 95% CI)	0.01 [-0.01, 0.02]
