# Supplementary figures and images for: Ivermectin for Prophylaxis and Treatment of COVID-19: A Systematic Review and Meta-Analysis
Source: Diagnostics (Basel). 2021 Sep 8;11(9):1645. doi: 10.3390/diagnostics11091645 (PMC8470309; doi:10.3390/diagnostics11091645)

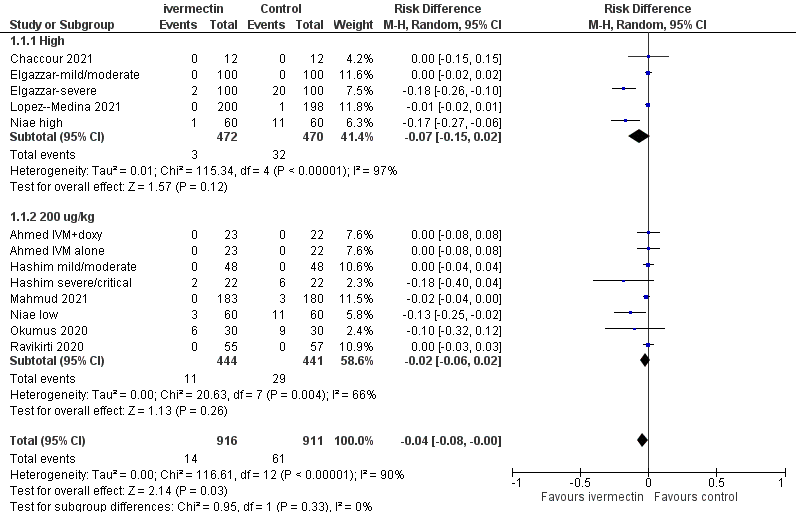

Supplement: Supplementary file 1 [file diagnostics-11-01645-s001.zip › Suppl. Figure S2.png]

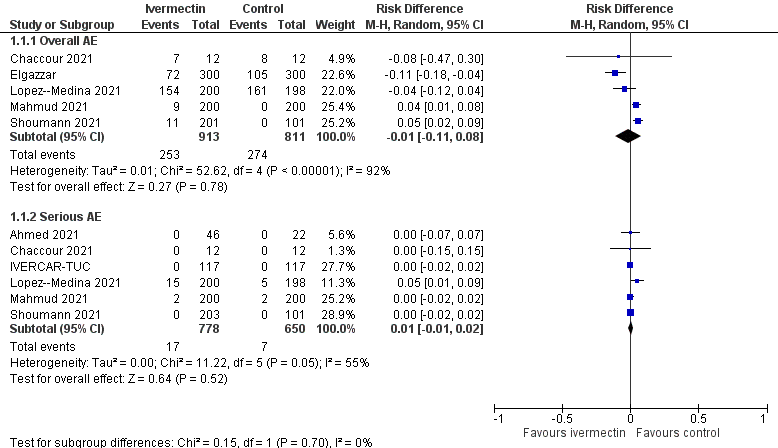

Supplement: Supplementary file 1 [file diagnostics-11-01645-s001.zip › Suppl. Figure S3.png]

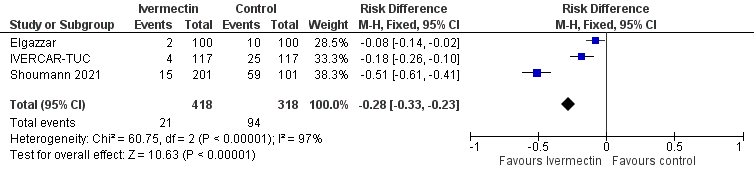

Supplement: Supplementary file 1 [file diagnostics-11-01645-s001.zip › Suppl. Figure S4.png]

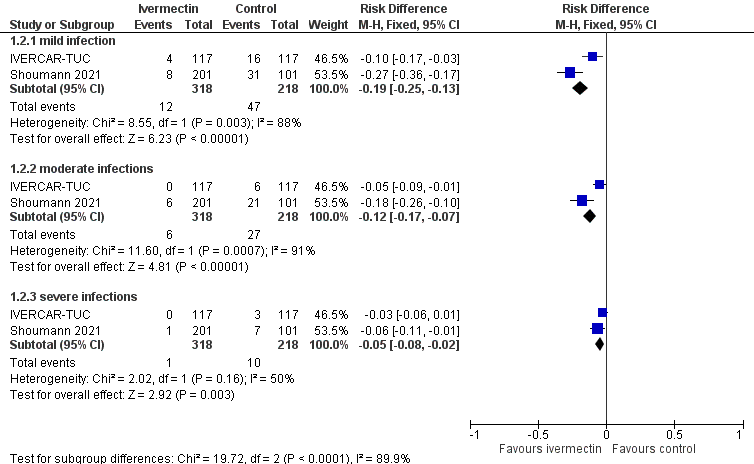

Supplement: Supplementary file 1 [file diagnostics-11-01645-s001.zip › Suppl. Figure S5.png]
